# Characterization of lipoteichoic acid structures from three probiotic *Bacillus* strains: involvement of d-alanine in their biological activity

**DOI:** 10.1007/s10482-014-0239-8

**Published:** 2014-08-05

**Authors:** Romain Villéger, Naima Saad, Karine Grenier, Xavier Falourd, Loïc Foucat, Maria C. Urdaci, Philippe Bressollier, Tan-Sothea Ouk

**Affiliations:** 1 Laboratoire de Chimie des Substances Naturelles, EA 1069, Antenne IUT, Département Génie Biologique, Allée André Maurois, 87065 Limoges, France; 2 UMR 1268 Biopolymères Interactions Assemblages, INRA, 44316 Nantes, France; 3 UMR 5248, Université de Bordeaux-Bordeaux Sci Agro, Microbiology Lab, 1 cours du General de Gaulle, 33175 Gradignan, France

**Keywords:** Lipoteichoic acid, Probiotics, Immunomodulation, Structure–activity relationship

## Abstract

Probiotics represent a potential strategy to influence the host’s immune system thereby modulating immune response. Lipoteichoic Acid (LTA) is a major immune-stimulating component of Gram-positive cell envelopes. This amphiphilic polymer, anchored in the cytoplasmic membrane by means of its glycolipid component, typically consists of a poly (glycerol-phosphate) chain with d-alanine and/or glycosyl substitutions. LTA is known to stimulate macrophages in vitro, leading to secretion of inflammatory mediators such as Nitric Oxide (NO). This study investigates the structure–activity relationship of purified LTA from three probiotic *Bacillus* strains (*Bacillus cereus* CH, *Bacillus subtilis* CU1 and *Bacillus clausii* O/C). LTAs were extracted from bacterial cultures and purified. Chemical modification by means of hydrolysis at pH 8.5 was performed to remove d-alanine. The molecular structure of native and modified LTAs was determined by ^1^H NMR and GC–MS, and their inflammatory potential investigated by measuring NO production by RAW 264.7 macrophages. Structural analysis revealed several differences between the newly characterized LTAs, mainly relating to their d-alanylation rates and poly (glycerol-phosphate) chain length. We observed induction of NO production by LTAs from *B. subtilis* and *B. clausii*, whereas weaker NO production was observed with *B. cereus*. LTA dealanylation abrogated NO production independently of the glycolipid component, suggesting that immunomodulatory potential depends on d-alanine substitutions. d-alanine may control the spatial configuration of LTAs and their recognition by cell receptors. Knowledge of molecular mechanisms behind the immunomodulatory abilities of probiotics is essential to optimize their use.

## Introduction

Beneficial effects of probiotic bacteria, notably immunomodulatory effects, have been attributed to cell envelope molecules interacting with intestinal cells (Lebeer et al. 2010). Among them, Lipoteichoic Acid (LTA) is a major constituent of Gram-positive bacteria cell envelopes and is considered as the counterpart of Gram-negative Lipopolysaccharide (LPS) (Weidenmaier and Peschel 2008; Wicken and Knox 1975). Many studies have described LTA to be an important factor for inflammation induced by pathogenic bacteria (Deininger et al. 2008; Ginsburg 2002), but also in immunomodulation mediated by probiotics. Although the nature of Pattern Recognition Receptors (PRR) involved in their recognition is still controversial (Ray et al. 2013), LTAs are described as ligands for TLR2 in a heterodimer with TLR6, with CD14 and CD36 as co-receptors (Nilsen et al. 2008; Schröder et al. 2003; Schwandner et al. 1999). Interaction between LTA and host cell PRR leads to induction of the NF-κB pathway, followed by production of pro-inflammatory mediators such as cytokines or Nitric Oxide (NO) (Hsiao et al. 2004; Kengatharan et al. 1996; Lebeer et al. 2012). However, the mechanisms underlying these effects are currently not well understood.

LTA is an amphiphilic glycopolymer, the most common structure of which in *Staphylococcus* and *Bacillus* strains is composed of a hydrophilic chain of poly (glycerol-phosphate) units, substituted with d-alanine and/or sugars, and covalently linked to β-gentiobiosyldiacylglycerol. This latter acts as a lipid anchor inserted into the plasma membrane, while the backbone chain extends into the peptidoglycan to the bacterial surface (Neuhaus and Baddiley 2003; Reichmann and Gründling 2011). Studies have shown variability in the LTA structure, depending on the bacterial strain. These variations, which mainly concern the length of the hydrophilic backbone (from 10 to 60 glycerol-phosphate units on average), the substitution rate with d-alanine (almost nothing to 80 % of the glycerol-phosphate units) and the fatty acid constituents of the hydrophobic anchor (13:0 in average). These different variations could modify the interactions with cell receptors and consequently, could lead to differences in the induced immunological responses. Currently, studies of structure–activity relationships are needed to highlight the exact role of each part of LTA in its biological activity. This has been mainly studied in *Staphylococcus aureus* given the involvement of this organism in sepsis, whereas LTAs from probiotic bacteria have been mostly described in *Lactobacillus* strains (Grangette et al. 2005; Mohamadzadeh et al. 2011; Vélez et al. 2007). LTAs from pathogenic *S. aureus* and probiotic *Lactobacillus plantarum* cause different immune-stimulatory effects. NO production induced by purified LTAs from the probiotic *L. plantarum* KCTC 10887BP in RAW 264.7 cells was reported to be lower than that from LTAs from pathogenic *S. aureus*, suggesting that their different effects on the immune system could be mainly caused by the distinctive structural features of their LTAs (Ryu et al. 2009). Multi-spectrometric analyses of LTA from *L. plantarum* KCTC 10887BP revealed major differences compared to *S. aureus* LTA, notably in the number and the degree of saturation of the acyl chain in the glycolipid moiety, and the types of modified sugar units in the poly (glycerol-phosphate) chains (Jang et al. 2011). Regarding the glycolipid anchor, it seems to play a critical role in biological activity of LTAs and related glycoconjugates (Baik et al. 2011; Blanc et al. 2013; Hong et al. 2014), notably because the acyl chain seems to be involved in recognition by cell receptor TLR2 (Kang et al. 2009), and necessary to induce expression of inflammatory components (Morath et al. 2002b). However, studies on the structural and functional relationship suggest that substitutions by d-alanine are mainly responsible for the immunostimulatory potential of LTAs. Beyond the role of d-alanine in cell viability, surface adhesion or protection against cationic antimicrobial peptides (Chan et al. 2007; Fittipaldi et al. 2008), Morath et al. (2001) demonstrated that a specific and complete hydrolysis of the d-alanine esters of *S. aureus* LTA led to a decrease in TNFα induction capacity in leukocytes from whole blood. Moreover, macrophage stimulation by *S. aureus* synthetic LTAs suggested that a strong cell response is dependent on a high degree of d-alanine substitutions (Deininger et al. 2003). The role of d-alanine was previously confirmed using an isogenic *L. plantarum* mutant with a complete absence of d-alanine substitution in LTA: significantly reduced levels of pro-inflammatory cytokines and enhanced levels of anti-inflammatory cytokines were observed compared to the parental strain in human Peripheral Blood Mononuclear Cells (PBMC) (Grangette et al. 2005).

Lactic acid bacteria and *Bifidobacteria* are the most commonly used bacterial probiotics. Strains of some *Bacillus* species have been shown to have prophylactic health benefits for human gastrointestinal disorders, prevention of recurrent respiratory infections or as an adjunct to antibiotic use (Cutting 2011; Hong et al. 2005; Marseglia et al. 2007; Mazza 1994). The use of such *Bacillus* strains as probiotics represents a real interest in dietary supplementation for their ability to induce immune stimulation and produce antimicrobial compounds, which is favoured by their ability to survive at a low gastric pH in a spore form (Cutting 2011; Urdaci et al. 2004). Furthermore, studies have shown that spores of probiotic *Bacillus* strains are able to germinate and grow within the intestinal tract, and possibly be considered as temporary residents (Casula and Cutting 2002; Duc et al. 2003; Leser et al. 2008). Effects on immunomodulation occur through the interaction and stimulation of Gut-associated Lymphoid Tissue (GALT), resulting in cytokine production. A previous study showed that the cell wall components from a probiotic *Bacillus* strain (*Bacillus coagulans* GBI-30) have immunomodulatory properties (Jensen et al. 2010).

In our study, we aimed to compare the structure and biological activity of LTAs from three *Bacillus* strains commercially sold as probiotic preparations for human use: *Bacillus cereus* CH (Anyang Yuanshou Biodrug Ltd., China), *Bacillus subtilis* CU1 (Probisis^®^BS, Lesaffre, France) and *Bacillus clausii* O/C (Enterogermina^®^, Sanofi Aventis, Italy). More specifically we aimed to determine the role of d-alanine substitution on the immunological activity of LTA. Purified LTAs were characterized by ^1^H NMR spectroscopy. LTA immunological activity was evaluated by measuring NO production as an indicator of inflammation using macrophage-like RAW 264.7 cells, and demonstrated that differences were linked to observed structural variations. Specific hydrolysis of d-alanyl ester bonds of LTAs was used to determine the involvement of d-alanine in the immunomodulatory potential of LTAs.

## Materials and methods

### Bacterial strains and growth conditions

*B. cereus* CH (Anyang Yuanshou^®^), *B. subtilis* CU1 (Probisis^®^) and *B. clausii* O/C (Enterogermina^®^) commercial probiotic strains were cultured in 2 L Erlenmeyer flasks at 37 °C in 400 mL Mueller–Hinton broth (Biokar Diagnostics, France) with shaking at 150 rpm.

### Extraction of LTA and hydrolysis of d-alanine esters

LTA extraction was adapted from Morath et al. (2001). Bacterial cells were grown to end log phase, in 400 mL cultures, and then harvested by centrifugation at 4,000×*g* for 15 min at 4 °C. Cells were washed three times with 0.1 M Tris–HCl buffer, pH 8 (Eurobio, France), then resuspended in 20 mL 0.1 M acetate buffer, pH 4.7 (Fisher Scientific, UK). The cells were mixed with an equal volume of *n*-butanol and incubated for 30 min at 37 °C under agitation at 300 rpm. After centrifugation at 13,000×*g* for 15 min at 4 °C, the aqueous phase was collected, and the bacterial pellet was resuspended again in 20 mL 0.1 M ammonium acetate buffer/pH 4.7 and disrupted by sonication to increase the amount of LTA (80 W, 2 min, Vibracell VC 750 Ultrasonic Processor, Hielscher, Germany). Following sonication, the cell suspension was stirred with an equal volume of *n*-butanol for 30 min at 37 °C with shaking at 300 rpm. After phase separation by centrifugation as described previously, the aqueous phases were pooled and lyophilized (VirTis, SP Scientific, USA).

In order to specifically hydrolyze the d-alanyl ester from the different *Bacillus* LTAs, a fraction of each extract was adjusted to pH 8.5 with NH_3_ and incubated overnight at 37 °C, as described previously (Morath et al. 2001). The dealanylation was monitored by ^1^H NMR.

### Purification of LTA

LTA purification was performed as described previously (Ryu et al. 2009). Briefly, dialysate was equilibrated with 0.1 M ammonium acetate buffer containing 15 % *n*-propanol (pH 4.7), then filtered and subjected to hydrophobic-interaction chromatography on an octyl-Sepharose HiPrep column (100 × 16 mm, GE Healthcare, U.K.). The mobile phase, composed of 15 % *n*-propanol in 0.1 M ammonium acetate buffer (pH 4.7), equilibrated the column and eluted unbound materials. LTA were eluted by a linear gradient of *n*-propanol from 15 % to 35 % *n*-propanol in 0.1 M ammonium acetate buffer (pH 4.7) at 2 mL/min. Collected fractions were analyzed on the basis of their phosphorus concentration as described below.

The LTA-containing fractions were subjected to DEAE-Sepharose anion-exchange chromatography (Fast Flow, GE with Healthcare, 100 × 10 mm column, Waters, USA) equilibrated in 20 mM ammonium acetate buffer (pH 4.7) with 30 % *n*-propanol. LTA was eluted using a 0 to 1 M sodium chloride linear gradient at 2 mL/min. Fractions collected containing LTA were pooled extensively and dialyzed against 500 volumes of 5 mM sodium acetate buffer (pH 4.7) using regenerated cellulose tubular membranes (molecular weight cut off: 3,500, Membrane Filtration Products, USA).

### Determination of phosphorus content

Phosphorus concentration was measured as described previously (Chen et al. 1956). For each fraction, 10 μL were mixed with 30 μL 10 % magnesium nitrate (w/v) in absolute ethanol, and were mineralized by flaming. The residue was dissolved with 0.3 mL 0.5 M HCl, then heated for 15 min at 100 °C. After cooling, 0.7 mL 0.42 % ammonium molybdate (v/v) in H_2_SO_4_:10 % ascorbic acid (w/v) 6:1 was added and the mixture was incubated at 37 °C for 1 h. Absorbance was determined with an appropriated standard at 820 nm (Libra S12, Biochrom, UK). The standard curve was performed using Na_2_HPO_4_.

### ^1^H NMR

NMR experiments were performed on an AVANCE III 400 Bruker spectrometer operating at a ^1^H frequency of 400.13 MHz. A double resonance ^1^H/X BBFo 5 mm probe was used. The acquisition pulse sequence used was a 90° pulse sequence with water presaturation and 10 s recycle time. Typically, the accumulation of 2,048 scans was used. Spectra were referenced using the 3-(trimethylsilyl) 3, 3, 2, 2-tetradeuteropropionic acid Na salt (d4-TSPA, Sigma-Aldrich, USA) signal at 0 ppm. FID signals were processed using Topspin 2.1^®^ software. A line broadening of 0.3 Hz was applied. Peakfit^®^ software was used for spectral deconvolution to access chemical shift, coupling constants and peak areas.

### LTA structural elucidation

Using chemical shifts previously assigned (Jang et al. 2011; Morath et al. 2001), data described in Table 1 were used for LTA structure elucidation. The average chain length of the poly (glycerol-phosphate) backbone and degree of d-alanine and *N*-acetylglucosamine substitutions were quantified from the results of Peakfit^®^ software deconvolution of LTA. The total amount of glycerol was determined from the area of the region between 3.8 and 4.2 ppm. The area ratio of glycerol and α-methylene group of the membrane anchor between 2.2 and 2.5 ppm identified the different average chain length. The area ratio of substitute alanine and total amount of glycerol gave the proportion of glycerol esterified by alanine. The area ratio of α-d-*N*-acetyl-glucosamine H_4_ (δ = 3.49 ppm) and total amount of glycerol gave the proportion of glycerol substituted by α-d-*N*-acetyl-glucosamine. The peak at 2.1 ppm assigned in the literature to *N*-acetyl wasn’t used for calculation because of the presence of superposed peaks at this chemical shift.

**Table 1:** ^1^H NMR chemical shift and coupling constant of *Bacillus* species lipoteichoic acid (LTA)

		δ (ppm)	^3^J (Hz)
Fatty acids (FA)	–CH_2_ (*α*)	2.35	–
	–CH_2_ (*γ*-*ω*)	1.30	–
	–CH_3_	0.88	–
Alanine (Ala)	Free H_*α*_	3.79	7.2
	Free H_*β*_	1.48	7.2
	Substituted H_*α*_	4.30	7.2
	Substituted H_*β*_	1.64	7.2
Glycerol	Total amount	[3.8–4.2]	–
*α*-d-*N*-acetyl-glucosamine (GN)	GN-H_4_	3.49	9.5
	GN-Ac	2.10	–

### Determination of fatty acid by GC–MS

Fatty acid composition was determined as described previously (Lepage and Roy 1986). Purified LTA (about 100 µg) was dried under vacuum with 10 μL of 10 mg/mL C17:0 used as an internal standard. The residue was dissolved in methanol/benzene/acetyl chloride 4:1:0,5 (v/v/v). After incubation at 100 °C for 1 h then cooling, 2.5 mL of 6 % K_2_CO_3_ (w/v) was added to the mixture. Tubes were centrifuged at 2,000 rpm for 5 min then 1 μL of supernatant loaded on the GC–MS system (gas chromatograph GC8060, mass spectrometer MD800, Fison Instrument, Italy). A capillary column INNOWAX (30 m × 0.25 mm i.d.) coated with 0.15 μm polyethyleneglycol film (Agilent J&W Scientific, USA) was used for separation and maintained at 180 °C in the oven. The supernatant was injected at 250 °C and eluted by high purity helium as carrier gas, with a 2 mL/min split mode flow rate (1:20).

### RAW 264.7 cells

The mouse macrophage cell line RAW 264.7 was obtained from the ATCC (TIB-71™) and was cultured in Roswell Park Memorial Institute Medium (RPMI, Gibco, USA) supplemented with 10 % heat inactivated fetal bovine serum (FBS, Gibco, USA), 2 mM glutamine (Gibco, USA), 1 % non-essential amino-acids, 1 % vitamins, 100 IU/mL penicillin (Gibco, USA), 100 µg/mL streptomycin (Gibco, USA) and 1 mM sodium pyruvate (Gibco, USA).The cells were cultured in a humid atmosphere, at 37 °C and 5 % CO_2_.

### NO production

Nitrite accumulation in the culture medium was considered to be an indication of NO production as described previously (Green et al. 1982). RAW 264.7 cells were seeded in 96-well plates at 1 × 10^4^ cells per well overnight. Cells were stimulated for 48 h with LTA from probiotic *Bacillus* strains at three concentrations (0.1, 1 and 10 µg/mL). 0.8 mL of Griess reagent (0.5 % sulfanilic acid, 1.4 % acetic acid, and 0.008 % naphtylethylenediamine) was mixed with culture supernatants. The optical density was determined at 535 nm (Libra S12, Biochrom, UK) by comparison with a NaNO_2_ standard curve. A negative control with 5 mM sodium acetate buffer (pH 4.7) and two positive controls with commercial LPS from *Escherichia coli* K12 (InvivoGen, France) and commercial LTA from *S. aureus* (InvivoGen) were used.

LTA cytotoxicity was assessed by adding 3-[4,5-dimethylthiazol-2yl]-2,5-diphenyltetrazolium bromide solution (MTT, Sigma-Aldrich, USA) at a final concentration of 0.5 mg/mL in each well for 3 h at 37 °C in a humidified atmosphere containing 5 % CO_2_. Afterwards, supernatants were removed and formazan crystals were dissolved with 200 µL DMSO. Absorbance was measured with a microplate spectrophotometer at 595 nm (TRIAD series Multimode Detector, Dynex, USA) and compared to controls (data not shown).

## Results & discussion

### ^1^H NMR and GC–MS analyses reveal structural variations between three *Bacillus* LTAs

**Fig. 1:**
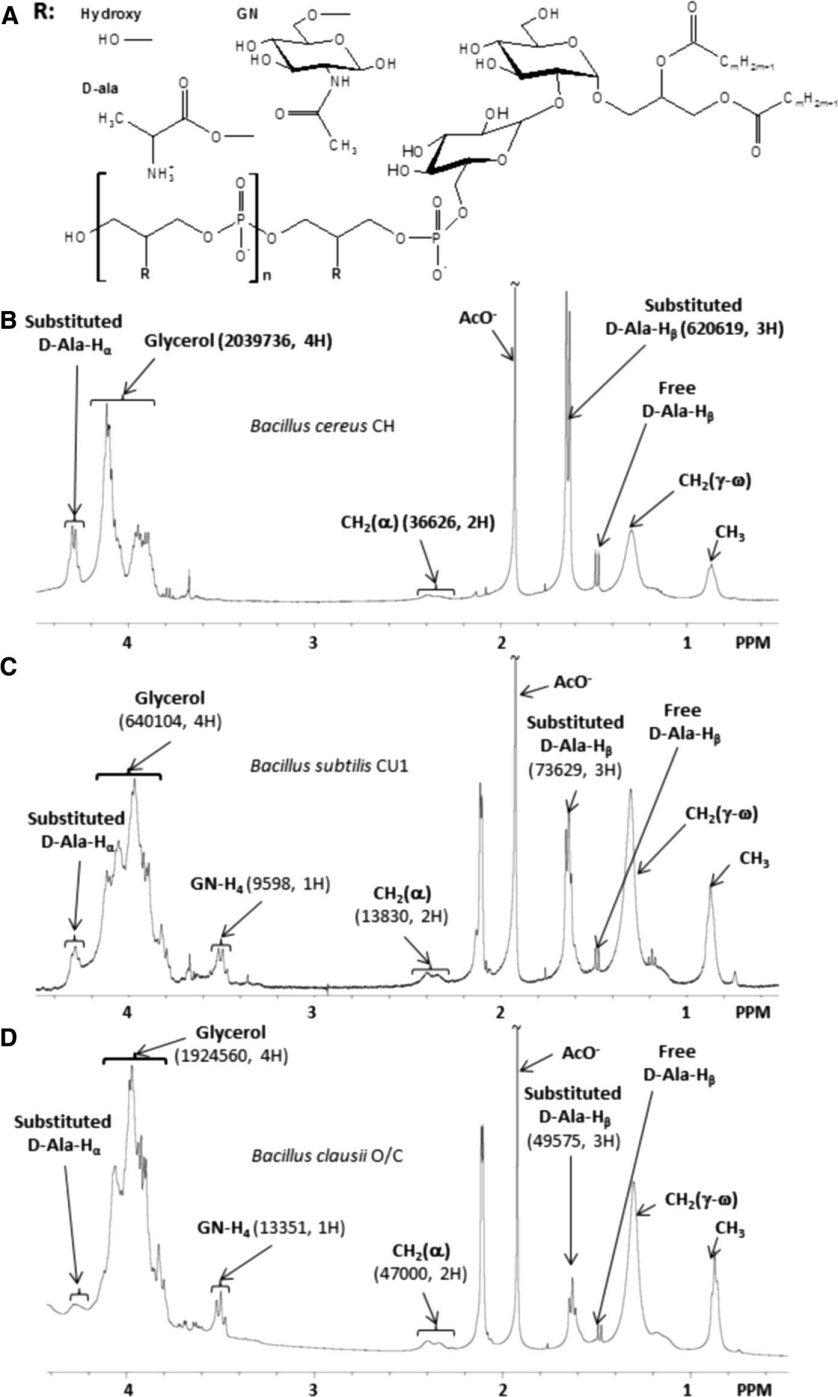
(a) General model of the molecular structure of *Bacillus* LTAs as determined from ^1^H NMR analysis. n represents the number of glycerol-phosphate repeating units of the hydrophilic backbone, and R the substituent group of glycerol. R can be hydroxyl groups (Hydroxy), d-alanine (d-Ala) or *N*-acetylglucosamine (GN). (b) ^1^H NMR spectrum of *B. cereus* CH LTA. (c) ^1^H NMR spectrum of *B. subtilis* CU1 LTA. (d) ^1^H NMR spectrum of *B. clausii* O/C LTA. Areas (in a.u.) and the corresponding number of hydrogen used for chain length, proportion of glycerol esterified with alanine and proportion of glycerol substituted with *N*-acetylglucosamine calculations, are indicated in bracket

In order to compare the structure of LTAs from three different *Bacillus* strains, they were extracted using the *n*-butanol method (Morath et al. 2001) and purified by hydrophobic-interaction chromatography followed by ion-exchange chromatography (Ryu et al. 2009). Chemical structures were assessed for each LTA using ^1^H NMR spectroscopy and results are shown in Fig. 1. ^1^H NMR spectra of LTAs obtained in previous studies (Jang et al. 2011; Morath et al. 2001; Wörmann et al. 2011) were used as references to assign the chemical shifts in our analysis (Table 1) and quantification is summarized in Table 2. Results presented in Fig. 1 and Tables 1, 2 and 3 are based on a single LTA isolation. LTA structural characterizations were also done on other preparations and showed comparable results (data not shown).

**Table 2:** Structure of native LTA prepared from *Bacillus* strains determined by ^1^H NMR spectroscopy (see paragraph 3.7)

	*B.cereus* CH	*B.subtilis* CU1	*B.clausii* O/C
Glycerol-phosphate chain length (n)	28	23	20
d-alanine (d-Ala) substitution rate	41 %	17 %	3 %
*N*-acetylglucosamine (GN) substitution rate	0 %	7 %	3 %
Estimated molecular weight (Da)	5,800	4,900	4,000

**Table 3:** Fatty acid composition of LTA, expressed in percentage of total fatty acids

Fatty acid	Percentage of total fatty acids		
	*B.cereus*	*B.subtilis*	*B.clausii*
	CH	CU1	O/C
C14:0	13	0	2.3
Iso- and Anteiso-C15:0	51	59.8	58.5
C16:0	10.3	9.4	9.5
Iso- and Anteiso-C16:0	11.4	8	9
C16:1	0	0	0
Iso-C17:0	9.1	8.5	8.1
Anteiso-C17:0	5.2	14.3	12.6
C18:0	0	0	0
C18:1	0	0	0

The ^1^H NMR spectra (Fig. 1) showed characteristic signals for glycerol between 3.8 ppm and 4.2 ppm, which were used to determine the number of (glycerol-phosphate) repeating units of each LTA molecule. Signal at δ = 3.45 ppm was used for determination of *N*-acetylglucosamine substitutions and d-alanine substitutions were quantified from signals at δ = 1.64 ppm and δ = 4.30 ppm (Table 1). The ^1^H NMR spectrum of *B. cereus* LTA indicated the longest poly (glycerol-phosphate) chain length with an average of 28 U, and the highest degree of d-alanyl substitutions (41 %) without glucosamine. Analysis of *B. subtilis* LTAs showed an average poly (glycerol-phosphate) chain length of 23 U, substituted with about 17 % d-alanine and 7 % *N*-acetylglucosamine. The hydrophilic backbone of *B. clausii* LTA, with a polymerization degree of 20, is substituted with 3 % *N*-acetylglucosamine and only 3 % d-alanine (Table 2). The results obtained for the poly (glycerol-phosphate) chain length are in agreement with a previous study on *Bacillus* LTAs (Iwasaki et al. 1989), who calculated the number of repeating units in the poly (glycerol-phosphate) chain of LTAs from several *Bacillus* strains to be 25–35. However, this determination was not investigated using spectroscopic analysis, and preparation of LTAs was done with the hot phenol extraction method. A previous NMR study on a commercial *B. subtilis* LTA (from *B. subtilis* DSMZ 1087) showed LTA with a short hydrophilic backbone (22 repeating units of glycerol-phosphate in average) (Morath et al. 2002a). In our study, despite differences in chemical composition of LTA depending on the strain, our observations seem to confirm the presence of a relatively short poly (glycerol-phosphate) backbone in LTA from several *Bacillus* species. However, (Morath et al. 2002a, b) found a substitution rate of 25 % with d-alanine and *N*-acetylglucosamine, which is quite different from that of our *B. subtilis* strain. This observation shows that the chemical composition of LTAs may vary within the same species. d-alanine substitution is the most variable part in the LTA structure of the three studied *Bacillus* strains, with a substitution rate of 17 % in *B. subtilis* LTA, 3 % in *B. clausii* LTA and 41 % in *B. cereus* LTA. These major differences could lead to different biological activities of the LTAs. Our results show a reverse distribution of d-alanine substituent compared to a previous study on *Enterococcus faecalis* (Kiel 27738) LTAs extracted using the hot-phenol method (Leopold and Fischer 1992). We observed that as the chain length increases, the d-alanine substitution rate increased as well. Chemical characterization of LTA from the pathogen *S. aureus* (strain DSM 20233) revealed a degree of polymerization ranging between 45 and 50, with a high substitution rate for d-alanine (70 %) and only 15 % *N*-acetylglucosamine (Morath et al. 2005). Compositional differences between *S. aureus* LTA and the described *Bacillus* LTAs of this study highlight significant variability in composition between LTAs from members of two distinct genera, and moreover between pathogenic and probiotic bacteria. Presumably these structural differences could be involved in recognition by eukaryotic cells to distinguish between pathogenic and non-pathogenic bacteria. *N*-acetylglucosamine substitutions in LTAs of the three *Bacillus* appeared less numerous than d-alanine substitutions. *B. cereus* LTA showed no substitution of hydrophilic chains with glucosamine, while we found only 3 % *N*-acetylglucosamine substitutions in poly (glycerol-phosphate) chains of *B. clausii* LTA and 7 % in *B. subtilis* LTA (Table 2). β-GlcNac-P-polyprenol synthetase or the enzyme catalyzing *N*-acetylglucosamine transfer from β-GlcNac-polyprenol to LTA could be responsible for the *N*-acetylglucosamine branches on poly (glycerol-phosphate) chains. However, the role of *N*-acetylglucosamine is not well understood because of the lack of studies and greater attention given to d-alanine. Moreover, estimation of the molecular weight of *Bacillus* LTAs using ^1^H NMR demonstrated their small size (4,000–5,800 Da), compared with those of *S. aureus* or *L. plantarum*, reflecting their shorter hydrophilic backbones (Table 2).

Regarding the lipid anchor of LTA, very few authors have analyzed the nature of fatty acids (Morath et al. 2001; Roethlisberger et al. 2000; Shiraishi et al. 2013). Previous structural characterizations of LTAs were done using NMR spectroscopy, but this way does not allow the determination of the nature of the acyl chains because of the microheterogeneity in the fatty acid composition. In order to describe the lipid anchor nature and determine more precisely the length of fatty acids, we performed a GC–MS analysis as described in Methods. Results are expressed as a percentage of each specified fatty acid to total fatty acids presented in purified LTAs (Table 3). LTA exhibited similar composition in fatty acids between the different *Bacillus* strains. These results showed the presence of branched fatty acids, mainly iso- and anteiso-C15:0, and in lesser amounts C14:0, C16:0, iso- and anteiso-C16:0, and iso- and anteiso-C17:0, which is in agreement with previous study on LTA from other *Bacillus* strains (Iwasaki et al. 1989). These branched fatty acids have been previously identified in the membranes of some bacterial genera including *Bacillus* (Li et al. 2010). However, the content of each fatty acid differed significantly between *Bacillus* strains, especially in *B. cereus* with lower amounts of anteiso-C17:0 and higher amounts of C14:0. Given the possible role of the lipid anchor in the LTA recognition by cell receptors (Jin et al. 2007), these structural variations could be partly responsible for differences in LTA biological activity but this needs to be investigated further.

### Levels of nitric oxide production by RAW 264.7 cells induced by LTAs is strain dependent

**Fig. 2:**
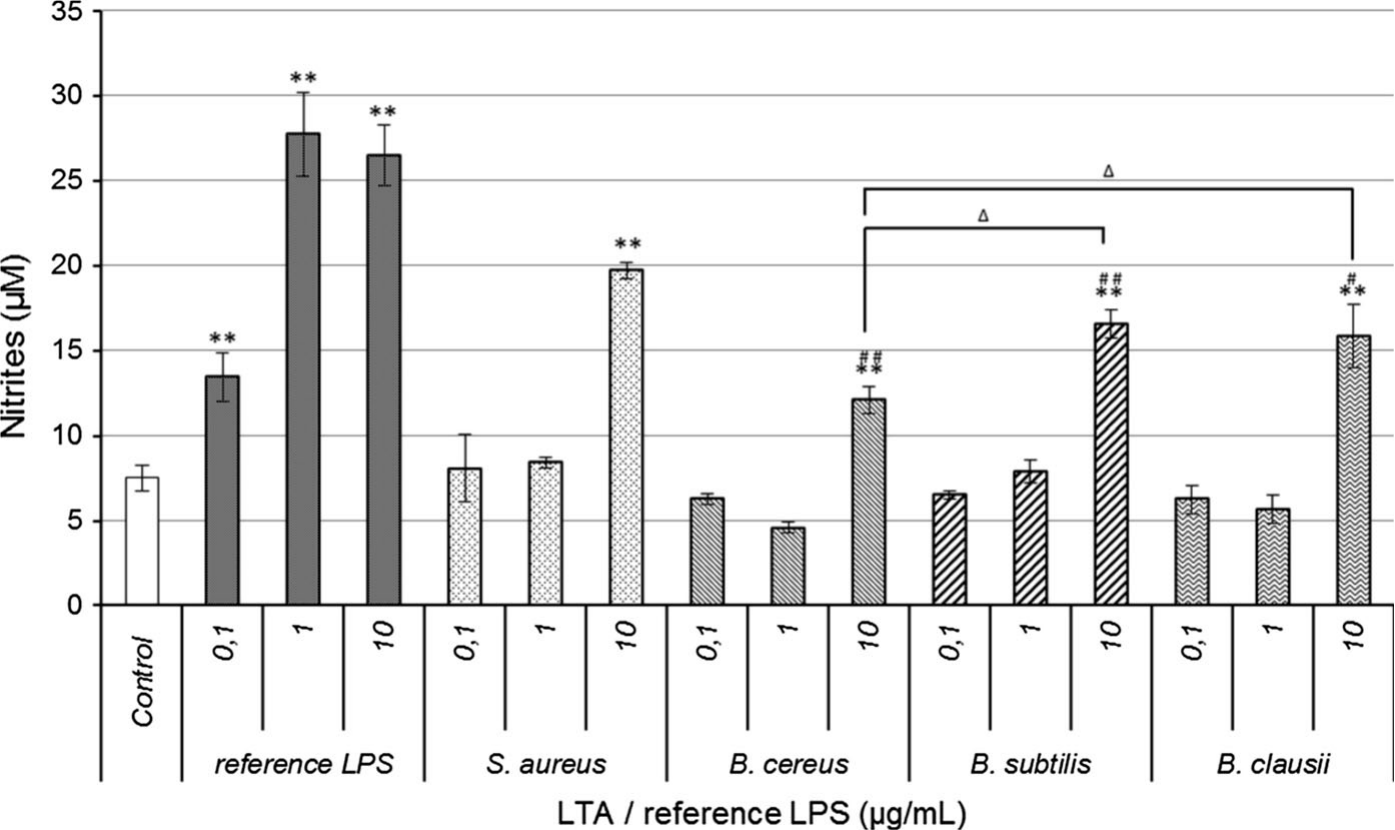
Probiotics *B. cereus*, *B. subtilis* and *B. clausii* LTA enhance NO production in RAW 264.7 cells, but in lesser amounts than LPS from pathogenic *E. coli* or LTA from *S. aureus*. RAW 264.7 cells (1 × 10^4^ cells per well) were incubated with indicated concentrations of LTA or reference LPS for 48 h, and nitrite production was measured in culture supernatants. Values are median ± S.D. of three replicates for each group. *p < 0.05; **p < 0.01 as compared with the control; ^∆^ *p* < 0.05 as compared with *B. cereus* LTA at 10 µg/mL; ^#^ *p* < 0.05; ^##^p < 0.01 as compared with commercial *S. aureus* LTA at 10 µg/mL

Among the beneficial effects of probiotic bacteria, their ability to modulate intestinal immune responses could be mediated by LTAs in Gram-positive organisms. Generation of NO is a key feature of many immune cells, including dendritic cells, NK cells, mast cells, and macrophages which recognize the molecular patterns associated with pathogens, e.g. LPS or LTA, via their PRR. NO has been shown to have diverse biological functions and considered as one of the most versatile players in the immune system. NO can induce an anti-inflammatory effect under normal and physiological conditions. However, NO is considered as a pro-inflammatory mediator that induces inflammation due to overproduction in abnormal situations. We investigated the potential of LTA from probiotic *Bacillus* strains to induce the immune defense mechanisms by testing their ability to enhance production of NO (Kang et al. 2011). RAW 264.7 cells were incubated with each highly purified LTA at three different concentrations (0.1, 1 and 10 µg/mL) for 48 h. The purity of each LTA was controlled by determination of amino acid composition (Bidlingmeyer et al. 1984) using the Pico-Tag Method (Waters, USA). No amino acids except d-alanine were present in the samples which confirmed the absence of contaminating lipoproteins (data not shown). Nitrite accumulation was determined as an indicator of NO production. Cell viability after treatment was monitored by MTT assay and showed no cytotoxicity (data not shown). Highly purified commercial LPS from *E. coli* K12 and LTA from *S. aureus* at the same concentrations were used as positive controls. To establish a rigorous correlation between structure and activity, biological assays were performed with the preparations previously characterized by ^1^H NMR and GC–MS. As shown in Fig. 2, NO production in the presence of *Bacillus* LTA or *S. aureus* LTA at 0.1 µg/mL and 1 µg/mL was similar to the negative control. However, at the same concentration, LPS was a stronger inducer of NO production. At 0.1 µg/mL, LPS induced NO production was almost two times greater than the negative control, and nearly three times higher at 1 µg/mL. These results are consistent with a previous report showing that LPS is more immunostimulatory than LTA for macrophages (Kimbrell et al. 2008). *S. aureus* LTA and each *Bacillus* LTA produced two fold more NO at 10 µg/mL than at lower concentrations, which led us to conclude that the NO-enhancing effects became significant at concentrations above 10 µg/mL LTA compared to the control. NO production observed with LTAs from *B. subtilis*, *B. clausii*, and *B. cereus* was significantly lower than that obtained with *S. aureus* LTA, although *B. subtilis* and *B. clausii* LTAs revealed their ability to induce high NO production. *B. cereus* LTA had lower inflammatory effects at the same concentration. We could hypothesize that these differences in the immunomodulatory properties of each LTA depend directly on their structure. Indeed, structural analysis showed that the lipid anchor composition of *B. cereus* LTA is significantly different to that of *B. subtilis* and *B. clausii* LTAs. It has been reported in several studies that the acyl fatty acid chains play a significant role in LTA-mediated immune response from pathogenic and beneficial bacteria, depending on their recognition by receptors (Baron and Kasper 2005; Han et al. 2003; Morath et al. 2002b). Although LTA from *Streptococcus pneumoniae* and *S. aureus* are structurally quite different, they were both previously described as ligands for TLR2. Pneumococcal LTA, the glycolipid anchor of which has unsaturated fatty acids, showed 100-fold less TLR stimulation than staphylococcal LTA consisting of only saturated fatty acids, as reported by Han et al. (2003). Although LTA recognition by TLR2 is actually under debate (Dammermann et al. 2013; Fischer et al. 2011; Ray et al. 2013; Schmidt et al. 2011), it is plausible from our study that the relatively low immunomodulatory effect of *B. cereus* LTA could be partially linked to the nature of the acyl chains. On the other hand, the d-alanine substitution rate of *B. cereus* LTA is the highest from the three studied strains (41 %), while this LTA was the less immunostimulatory. LTAs from *B. subtilis* and *B. clausii,* with a d-alanine substitution rate of 17 % and 3 % respectively, induced higher NO production. These results indicate that there is no proportional correlation between the substitution rate by d-alanine and NO production. Although *S. aureus* LTAs have been described with an approximately 70 % d-alanylation rate and high immunostimulatory potential, results obtained from our structure and activity study of *Bacillus* LTAs did not show that more alanine means higher activity.

Previous studies have shown that LTA from probiotic bacteria which induce NO production, but in less amounts than LPS, seems to have an inflammatory potential (Ryu et al. 2009). LTAs from the three studied strains could have the same properties, but it is not incompatible with a probiotic activity. There are two distinct types of immunomodulatory effects associated with the probiotic consumption, and which depend directly on the patients health (Heyman and Heuvelin 2006). The first is the ability of probiotics to reduce inflammation in patients with inflammatory bowel diseases, and the second is that probiotics could have an immunostimulatory effect on healthy individuals. This last point suggests that a probiotic, which interacts with intestinal cells, could lead to the secretion of pro-inflammatory compounds such as cytokines or NO. Inflammation induced by pro-inflammatory compounds is a helpful process in activating the host immune system against invading microbes. However, excessive inflammation can cause severe inflammatory diseases. *S. aureus* is a common pathogen involved in such pathologies and its LTA is a potent inducer of inflammation (Ginsburg 2002). LTA from the three *Bacillus* strains are weaker inducer of NO production compared to LPS or *S. aureus* LTA, which suggests that they could stimulate the immune system, but not to the point of leading to a pathologic level of inflammation as caused by LTA from pathogens. This hypothesis, previously proposed for *L. plantarum* (KCTC10887BP) LTA (Kim et al. 2008), is supported by the fact that the *Bacillus* strains studied here are described as beneficial immune stimulators.

Our results raise the question of the involvement of d-alanine in the biological activity of LTAs. To investigate the key role of d-alanine in the immunological activity of LTAs, each of them were specifically dealanylated and their NO induction potential was assessed.

### Dealanylation of *Bacillus* LTAs abrogates their ability to induce NO production by RAW 264.7 cells

**Fig. 3:**
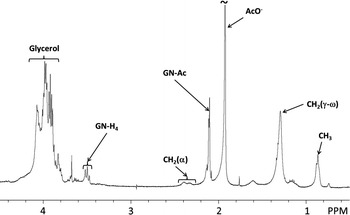
Efficiency of LTA chemical dealanylation controlled by ^1^H NMR analysis (example shown for *B. subtilis* CU1 LTA). d-alanine ester specific hydrolysis was assessed at pH 8.5 during 24 h (Morath et al. 2001)

**Fig. 4:**
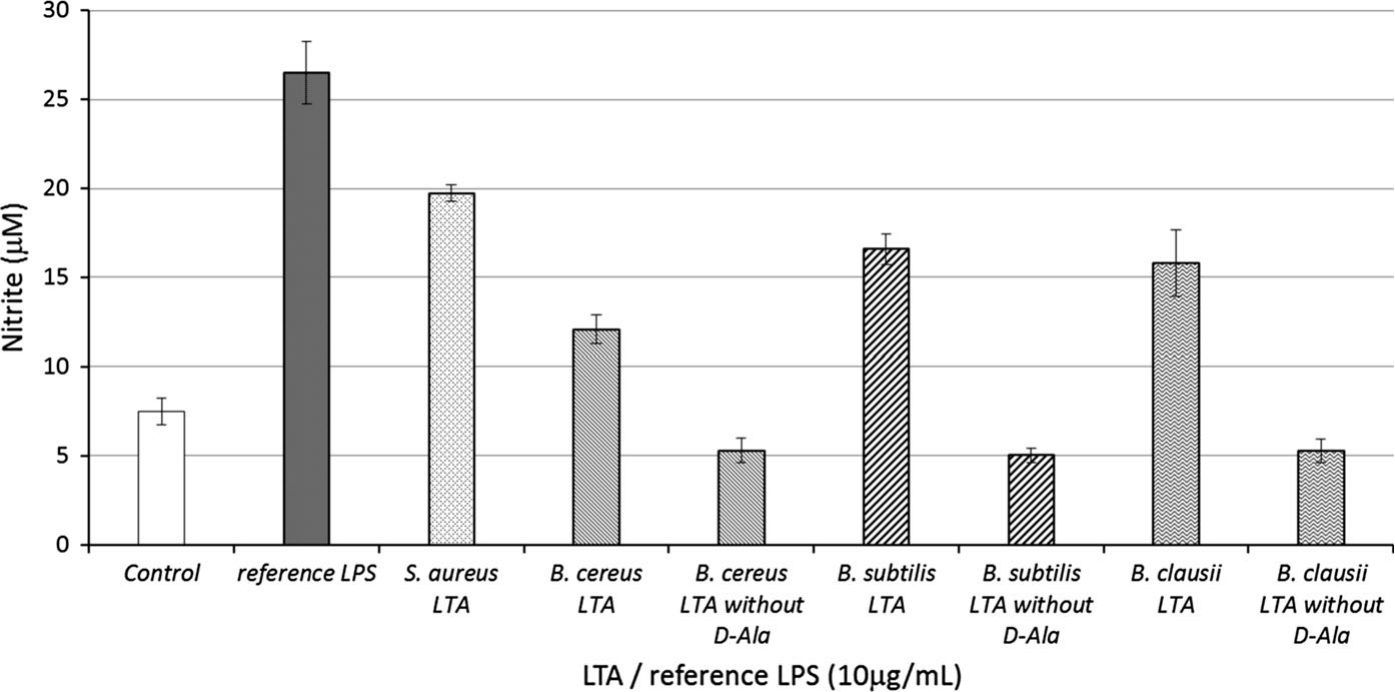
Hydrolysis of LTA d-alanine esters leads to LTA inactivation in NO production. d-alanine esters were hydrolyzed at pH 8.5 for 24 h. RAW 264.7 cells (1 × 10^4^ cells per well) were stimulated with 10 µg/mL *Bacillus* LTA, reference *S. aureus* LTA or reference LPS for 48 h. Nitrite production was measured as described previously (Green et al. 1982). Values are median ± S.D. of three replicates for each group

We next examined the effect of dealanylation on LTA immunological activity. Specific treatment of d-alanine ester could lead to the removal of all ester-bound substituents (Gisch et al. 2013). Previous structure/function relationship studies using partially degraded LTA tried to highlight structural features involved in LTA biological activity. However, these chemical modifications of LTA structure were not always specific and needed spectroscopic characterization to confirm the efficiency and specificity of the degradations before determining the biological activity. Here, each *Bacillus* LTA was treated at pH 8.5 for 24 h as described previously (Morath et al. 2001), resulting in hydrolysis of d-alanine esters. Efficiency and specificity of dealanylation was monitored by ^1^H NMR, with the loss of signals at δ = 1.64 ppm and δ = 4.30 ppm only. *B. subtilis* LTA dealanylation is shown as an example in Fig. 3 (results presented in Fig. 3 are based on a single LTA isolation). RAW 264.7 cells were incubated with dealanylated LTAs at 10 µg/mL for 48 h, followed by analysis of NO production (Fig. 4). Dealanylation completely abrogated the immunostimulatory potential of LTAs regardless of the *Bacillus* strain. Despite the controversial role of d-alanine observed in previous studies (Cot et al. 2011), our findings demonstrate that a baseline level of d-alanine substitution is fundamental to the biological activity of LTA, but that superfluous substitution is not directly proportional to immunological activity. Several hypotheses have to be considered regarding the potential role of d-alanine. Indeed, at a pH near seven, the d-alanine primary amino groups and phosphates of hydrophilic backbones are respectively cationic and anionic. A previous ^31^P NMR analysis of *Lactobacillus fermentum* NCTC 6375 LTA showed that phosphorus had two additional peaks in addition to the primary resonance which seemed to originate from differences in ion pairing between the amino function of d-alanyl residues and the two adjacent anionic phosphodiester anionic linkages (Batley et al. 1987; Neuhaus and Baddiley 2003). Neuhaus and Baddiley (2003) proposed two energy-minimized models, one in which the protonated amino group forms ion pairs with the upchain phosphodiester and in the one other with the down-chain phosphodiester. These intramolecular interactions lead to a decreased net anionic charge of LTA, and therefore an enhanced hydrophobic effect which could cause intramolecular folding. However, as observed previously (Garimella et al. 2009), this model disagrees with experimental data which show that the presence of d-alanine in the cell wall of Gram-positive bacteria provides resistance to cationic antimicrobial peptides through charge repulsion mechanisms (Peschel 2002). However, we hypothesize that ionic bonds created between certain d-alanines and non-adjacent phosphates, could lead to other three dimensional conformations. These two potential refoldings may limit intermolecular interactions of LTAs and could radically change their biological activity. It is important to consider the fact that various spatial conformations are not recognized in the same way by receptors and could lead to different biological responses by cells. Indeed, these differences in chemical composition of LTAs could modify their ability to interact with receptor binding sites. An earlier study on the interaction between LTA and the Macrophage Scavenger Receptor reported that a partial compensation for the negative charges of the hydrophilic chain with d-alanine decreased affinity for the receptor (Greenberg et al. 1996). A higher density of negative charges on LTAs probably plays an important role in affinity for PRR, but our study suggests that this is not what is causing their biological activity. Moreover, Nilsen et al. (2008) have demonstrated, in the case of TLR2 recognition, that blocking CD36 or CD14 inhibits binding of purified LTA to monocytes, which confirms the key role of co-receptors in the biological activity of LTA. From the results, we could suppose that the LTA lipid anchor is probably recognized by TLR2, but the hydrophilic chain could be involved in heterodimerization with TLR6 and/or recruitment of co-receptors. These observations are valid in the case of isolated molecules. However, we suppose that in solution, multimerization between two or more LTA molecules is possible. In the same manner as described previously, protonated amino groups of the LTA hydrophilic backbone could form ion pairs with the phosphodiester of other LTA molecules, leading to aggregation of hydrophilic chains and possibly to micellar arrangements (Fischer et al. 1997; Wicken et al. 1986). We can suppose that LTA dimerization may enhance their biological activity. It is also possible that hydrophilic chains of LTAs interact by hydrophobic effects in the case of intramolecular ion pairing, leading to other possibilities for intermolecular interactions. For these reasons, the spatial conformation of LTA must be studied as well as interactions between LTA and PRR. Molecular arrangements could be studied by dynamic molecular calculations. Different substitution rates of d-alanine and multimerisation of monomers must be considered to determine a model of hypothetical interactions.

## Conclusion

This study is the first which compared the structure and biological activity of LTA from probiotic strains of members of the genus *Bacillus*. The three studied *Bacillus* LTAs showed chemical composition differences, regarding both poly (glycerol-phosphate) chain length and the substitution rate with d-alanine and *N*-acetylglucosamine. Despite major differences in their structures, *B. subtilis* and *B. clausii* LTAs showed nearly the same ability to induce NO production by RAW 264.7, while *B. cereus* LTA seems to induce less by comparison. A key role for d-alanine in the macrophage stimulation activity of *Bacillus* LTA has been shown here. Using a strategy to modify a structural component of the bacterial cell envelope, we have contributed to the understanding of their potential role in immunomodulatory activity of probiotic *Bacillus* strains.
